# Aplicações de RNA Funcional na Medicina de Precisão Cardiovascular: Avanços e Perspectivas Diagnósticas

**DOI:** 10.36660/abc.20250553

**Published:** 2025-10-13

**Authors:** Mariana Gatto, Gustavo Augusto Ferreira Mota, Luana Urbano Pagan, Marina Politi Okoshi

**Affiliations:** 1 Universidade Estadual Paulista Júlio de Mesquita Filho Faculdade de Medicina Botucatu SP Brasil Universidade Estadual Paulista Júlio de Mesquita Filho - Câmpus de Botucatu - Faculdade de Medicina, Botucatu, SP – Brasil

**Keywords:** RNA, Circulação Coronária, Diagnóstico

Nos últimos anos, avanços em ferramentas diagnósticas têm revolucionado a pesquisa biomédica especialmente no que tange à identificação e compreensão de diversas doenças. Tecnologias de alta precisão, como o sequenciamento de última geração (*Next-Generation Sequencing*, NGS), aliadas às plataformas de bioinformática possibilitaram análise detalhada de perfis genéticos e transcriptômicos.^1^ Esse progresso viabilizou a detecção de variantes genéticas, assinaturas moleculares e padrões de expressão gênica que permitem refinar o diagnóstico de doenças complexas e identificar novos alvos terapêuticos.^2^

Dentre as tecnologias, destaca-se a RNA-Seq, uma aplicação do NGS voltada ao sequenciamento e quantificação de transcritos de RNA. Os transcritos podem ser classificados em RNAs codificantes, como os RNAs mensageiros (mRNAs), que direcionam a síntese proteica, e RNAs não codificantes, como os long non-coding RNAs (lncRNAs) e os microRNAs (miRNAs), que desempenham papéis regulatórios na expressão gênica.^3^ Os lncRNAs modulam a atividade transcripcional, epigenética e pós-transcricional de genes-alvo, enquanto os miRNAs em geral promovem a degradação de mRNAs ou inibem sua tradução.^4^ A análise da expressão de RNAs e de suas interações moleculares tem expandido o entendimento de redes de regulação gênica, com aplicações promissoras na oncologia e em doenças inflamatórias e cardiovasculares.^5^

O fenômeno do fluxo coronariano lento é caracterizado por retardo na perfusão de artérias coronárias distais na ausência de doença arterial coronariana obstrutiva.^6^ Embora a fisiopatologia do fenômeno não esteja completamente elucidada, evidências apontam para a contribuição de fatores de risco como gênero masculino, tabagismo, obesidade, alterações metabólicas e inflamação sistêmica.^7^ Estudos em biopsias miocárdicas têm mostrado doença microvascular e aumento do tono vasomotor coronariano em repouso.^8^

Clinicamente, indivíduos com fluxo coronariano lento podem apresentar angina em repouso ou durante esforço físico, arritmias e outros eventos cardiovasculares que comprometem a qualidade de vida e o prognóstico.^9^ Técnicas baseadas em sequenciamento gênico representam estratégia promissora para a descoberta de biomarcadores moleculares associados ao fluxo coronariano lento, que podem contribuir para o diagnóstico precoce e a estratificação de risco.

Recentemente, estudos mostraram desregulação de RNAs não codificantes em pacientes com fluxo coronariano lento. Alterações na expressão de lncRNAs como ANRIL, associado a ativação da via inflamatória NF-κB, e repressão de MALAT1 e LINC00305, ligados à disfunção endotelial, foram observadas em células sanguíneas.^10^ Nesta edição dos ABC, Jiang et al.^11^ identificaram por RNA-Seq 854 lncRNAs diferencialmente expressos em células mononucleares do sangue periférico de pacientes com fluxo coronariano lento. A análise de enriquecimento funcional revelou a participação de vias envolvidas em autofagia, degradação proteassomal e inflamação, incluindo os receptores NOD-like, TNF, toll-like e NF-κB. Complementarmente, Zhu et al.^12^ mostraram que sICAM-1, miR-148b-3p e NEAT1 atuam como preditores moleculares neste fenômeno. O lncRNA NEAT1, ao ligar-se ao miR-148b-3p, impede sua função reguladora sobre ICAM-1, aumentando a proliferação de células endoteliais — um mecanismo potencialmente associado a disfunção microvascular.

Diante do cenário emergente da medicina de precisão, a integração de tecnologias como RNA-Seq e análise bioinformática tem se mostrado fundamental para o entendimento de fenômenos cardiovasculares complexas como o fluxo coronariano lento. A identificação de redes regulatórias envolvendo lncRNAs e miRNAs oferece nova perspectiva para o diagnóstico precoce e o desenvolvimento de terapias direcionadas, fortalecendo o papel de biomarcadores moleculares como pilares da investigação clínica contemporânea.
