# Supplementary material for: Aplicações de RNA Funcional na Medicina de Precisão Cardiovascular: Avanços e Perspectivas Diagnósticas
Source: Arq Bras Cardiol. 2025 Oct 13;122(9):e20250553. [Article in Portuguese] doi: 10.36660/abc.20250553 (PMC12674905; doi:10.36660/abc.20250553)
Supplement: Translation

# Translation: Functional RNA Applications in Cardiovascular Precision Medicine: Advances and Diagnostic Perspectives

**DOI:** 10.36660/abc.20250553i

**Authors:** Mariana Gatto, Gustavo Augusto Ferreira Mota, Luana Urbano Pagan, Marina Politi Okoshi

**Affiliations:** 1 Botucatu SP Brazil Universidade Estadual Paulista Júlio de Mesquita Filho - Câmpus de Botucatu - Faculdade de Medicina, Botucatu, SP – Brazil

**Keywords:** RNA, Coronary Circulation, Diagnosis

In recent years, advancements in diagnostic tools have revolutionized biomedical research, particularly in the identification and understanding of various diseases. High-precision technologies such as Next-Generation Sequencing (NGS), coupled with bioinformatics platforms, have enabled detailed analyses of genetic and transcriptomic profiles.^1^ This progress has facilitated the detection of genetic variants, molecular signatures, and gene expression patterns, thereby refining the diagnosis of complex diseases and unveiling novel therapeutic targets.^2^

Among these technologies, RNA sequencing (RNA-Seq) —an NGS-based approach for sequencing and quantifying RNA transcripts—has emerged as a key tool. Transcripts can be classified into coding RNAs, such as messenger RNAs (mRNAs) that guide protein synthesis, and non-coding RNAs, including long non-coding RNAs (lncRNAs) and microRNAs (miRNAs), which play regulatory roles in gene expression.^3^LncRNAs modulate transcriptional, epigenetic, and post-transcriptional activity of target genes, whereas miRNAs generally promote mRNA degradation or inhibit translation.^4^ The analysis of RNA expression and molecular interactions has broadened our understanding of gene regulatory networks, with promising applications in oncology, inflammatory diseases, and cardiovascular conditions.^5^

The coronary slow flow phenomenon is characterized by delayed perfusion of distal coronary arteries in the absence of obstructive coronary artery disease.^6^ Although its pathophysiology is not yet fully elucidated, evidence suggests contributions from risk factors such as male sex, smoking, obesity, metabolic alterations, and systemic inflammation.^7^ Studies on myocardial biopsies have demonstrated microvascular disease and increased resting coronary vasomotor tone.^8^

Clinically, individuals with coronary slow flow may present with resting or exertional angina, arrhythmias, and other cardiovascular events that impair quality of life and prognosis.^9^ Gene sequencing–based techniques represent a promising strategy for the discovery of molecular biomarkers associated with coronary slow flow, potentially enabling early diagnosis and risk stratification.

Recent studies have reported dysregulation of non-coding RNAs in patients with coronary slow flow. Altered expression of lncRNAs such as ANRIL, associated with activation of the NF-κB inflammatory pathway, and downregulation of MALAT1 and LINC00305, linked to endothelial dysfunction, have been observed in blood cells.^10^ In this edition of *Arquivos Brasileiros de Cardiologia (ABC)*, Jiang et al.^11^ identified, via RNA-Seq, 854 differentially expressed lncRNAs in peripheral blood mononuclear cells from patients with coronary slow flow. Functional enrichment analysis revealed the involvement of pathways related to autophagy, proteasomal degradation, and inflammation—including NOD-like receptors, TNF, toll-like receptors, and NF-κB. Complementarily, Zhu et al. ^12^ demonstrated that sICAM-1, miR-148b-3p, and NEAT1 act as molecular predictors in this phenomenon. The lncRNA NEAT1, by binding to miR-148b-3p, inhibits its regulatory function on ICAM-1, promoting endothelial cell proliferation—a mechanism potentially associated with microvascular dysfunction.

In the emerging landscape of precision medicine, the integration of technologies such as RNA-Seq and bioinformatics analysis has proven fundamental to understanding complex cardiovascular phenomena such as coronary slow flow. The identification of regulatory networks involving lncRNAs and miRNAs offers a new perspective for early diagnosis and the development of targeted therapies, reinforcing the role of molecular biomarkers as cornerstones of contemporary clinical investigation.
